# High Mortality of HLH in ICU Regardless Etiology or Treatment

**DOI:** 10.3389/fmed.2021.735796

**Published:** 2021-10-06

**Authors:** Amandine Bichon, Jérémy Bourenne, Jérôme Allardet-Servent, Laurent Papazian, Sami Hraiech, Christophe Guervilly, Vanessa Pauly, Gilles Kaplanski, Djamel Mokart, Marc Gainnier, Julien Carvelli

**Affiliations:** ^1^APHM, University Timone Hospital, Réanimation des Urgences, Marseille, France; ^2^Aix-Marseille University, Marseille, France; ^3^Department of Intensive Care, Européen Hospital, Marseille, France; ^4^Department of Respiratory and Infectious Intensive Care, APHM, University Nord Hospital, Marseille, France; ^5^Department of Medical Information, CEReSS - Health Service Research and Quality of Life Center, APHM, Aix-Marseille University, Marseille, France; ^6^Department of Internal Medicine and Clinical Immunology, APHM, University Conception Hospital, Marseille, France; ^7^Department of Onco-Hematological Intensive Care, Paoli Calmette Institute, Marseille, France

**Keywords:** hemophagocytic lymphohistiocytosis, macrophage activation syndrome, hemophagocytosis, cytokine storm, intensive care unit

## Abstract

**Background:** Adult hemophagocytic lymphohistiocytosis (HLH) is highly lethal in the ICU. The diagnostic and therapeutic emergency that HLH represents is compounded by its unknown pathophysiological mechanisms. Here, we report on a large cohort of adult HLH in the ICU (ICU-HLH). We analyzed prognostic factors associated with mortality to define the diagnostic and therapeutic challenges in this specific population.

**Methods:** This retrospective study included adult patients diagnosed with HLH in four ICUs in Marseille, France between 2010 and 2020. Patients who fulfilled the HLH-2004 criteria (≥ 4/8) and/or had an HScore ≥ 169 were diagnosed with HLH. HLH was categorized into four groups according to etiology: sepsis-associated HLH, intracellular infection-associated HLH, malignancy-associated HLH, and idiopathic HLH.

**Results:** Two hundred and sixty patients were included: 121 sepsis-associated HLH (47%), 84 intracellular infection-associated HLH (32%), 28 malignancy-associated HLH (11%), and 27 idiopathic HLH (10%). The ICU mortality rate reached 57% (*n* = 147/260) without a statistical difference between etiological groups. Independent factors associated with mortality in multivariate analysis included age (OR (5 years) = 1.31 [1.16–1.48], *p* < 0.0001), SOFA score at ICU admission (OR = 1.37 [1.21–1.56], *p* < 0.0001), degradation of the SOFA score between ICU arrival and HLH diagnosis (Delta SOFA) (OR = 1.47 [1.28–1.70], *p* < 0.0001), the presence of bone-marrow hemophagocytosis (OR = 5.27 [1.11–24.97], *p* = 0.04), highly severe anemia (OR = 1.44 [1.09–1.91], *p* = 0.01), and hypofibrinogenemia (OR = 1.21 [1.04–1.41], *p* = 0.02).

**Conclusions:** In this large retrospective cohort study of critically ill patients, ICU-HLH in adults was associated with a 57% mortality rate, regardless of HLH etiology or specific treatment. Factors independently associated with prognosis included age, presence of hemophagocytosis in bone-marrow aspirates, organ failure at admission, and worsening organ failure during the ICU stay. Whether a rapid diagnosis and the efficacy of specific therapy improve outcome is yet to be prospectively investigated.

## Take Home Message

ICU-HLH in adults was associated with a 57% mortality rate, regardless of HLH etiology or etoposide administration. Factors independently associated with prognosis included age, the presence of hemophagocytosis in bone-marrow aspirates, organ failure at admission, and worsening organ failure during the ICU stay.

## Introduction

Adult hemophagocytic lymphohistiocytosis (HLH) can be defined as the most extreme form of the inflammatory process continuum. Imbalances in or a failure of feedback between pro- and anti-inflammatory pathways in response to a trigger lead to uncontrolled macrophage/monocyte and lymphocyte activation and proliferation (1). A sustained cytokine storm may complicate HLH syndrome, ultimately leading to multiorgan dysfunction (MODS) (2, 3). Diagnostic criteria for HLH include clinical parameters (fever, adenopathy, splenomegaly, hepatomegaly) and biological variables (cytopenia, hyperferritinemia, hypertriglyceridemia, hypofibrinogenemia), although these criteria are non-specific and may be inappropriate, as they are extrapolated from the pediatric population (4). Pediatric primary HLH is mostly caused by genetic mutations in genes involved in lymphocyte cytotoxicity (5–8). In contrast, the analysis of adult HLH has highlighted the absence of a lymphocyte cytotoxicity defect (9). The first step for critically ill patients is to distinguish most difficult part in a critically ill patients is thus the hypothesis and confirmation of HLH diagnosis.

As adult-onset HLH constitutes a post-trigger inflammatory process, pathophysiological clusters can be described, mirroring their etiology (10, 11): infection-associated HLH (bacteria, Herpesviridae, mycobacteria, parasites, fungi), malignancy-associated HLH, and systemic auto-immune/inflammatory-disease-associated HLH (systemic erythematosus lupus, juvenile chronic arthritis, adult Still disease), the latter still defined as macrophage-activation syndrome (1, 10). Careful etiological diagnosis must be performed promptly, as it has a large influence on the prognosis and defines treatment (12). Understanding the pathophysiology underlying HLH is the key to cytokine-storm and/or cellular-proliferation targeted therapy (13, 14). However, literature does not distinguish specific etiology and assembles HLH triggers with different pathophysiological patterns (stable vs. inaugural cancer or bacterial vs. viral triggers for instance).

The diagnosis of HLH in ICU patients is often difficult, presenting as “hyper-inflammatory sepsis” (15). Sepsis is often described as the trigger for HLH in ICU patients (16, 17). Without any obvious etiology, such as cancer, the diagnosis of acquired HLH leads to uncertainty concerning the decision to initiate specific treatment. Therapy for ICU-HLH mainly involves standard organ support, thorough etiological screening, and urgent treatment of the HLH trigger (infection, auto-immune underlying condition, malignant process). “Specific” HLH therapies (corticosteroids, polyvalent immunoglobulins, anti-cytokine therapies, etoposide) are often solely administered in the severest cases of ICU-HLH. Targeted therapy is based on the HLH-2004 protocol (18, 19). However, acquired adult HLH and primary pediatric HLH constitute the endpoints of a continuous spectrum of the disease, with non-comparable pathophysiology although overlapping forms have been described (9, 20). In addition, systematic immunosuppressive therapy on fragile ICU patients could aggravate their condition and expose them to a high risk of infection (21). Finally, cytotoxic therapies against HLH have not been studied according to HLH etiology.

We aimed to describe the epidemiological, clinical, and biological characteristics of ICU-HLH in adults according to etiological cluster to better understand the disease and aid in its rapid diagnosis and delivery of appropriate treatment. By defining the prognostic factors associated with ICU mortality, we focus on patients for whom their condition constitutes a diagnostic and therapeutic emergency.

## Methods

We performed a retrospective observational multi-institutional study for patients admitted to the ICUs of the European Hospital, Timone University Hospital, and Nord University Hospital and the Onco-Hematological ICU of the Paoli-Calmette Institute, in Marseille, France between January 2010 and June 2020. The medical information department (DIM) of each hospital provided the cohort using the following keywords: “hemophagocytosis”, “hemophagocytic lymphohistiocytosis”, “macrophage-activation syndrome” and “hyperferritinemia”. Only adult patients with ICU-HLH were included among the selected files. An HLH diagnosis required fulfillment of at least four of eight HLH-2004 criteria [95% sensitivity and 93.6% specificity for ICU adult patients (22)] and/or an HScore ≥ 169 [93% sensitivity and 86% specificity (23)]. A HLH-2004 threshold at 4/8 instead of 5/8 has recently proven to be more accurate for ICU-HLH diagnosis, higher scores only being associated with higher mortality rates but not better diagnosis (22). This study was communicated to the Commission on Data Processing and Freedom representative of each center and approved by APHM ethic committee (#2019-316). Immunosuppression was reported for stem-cell or solid-organ transplantation, solid-organ cancer, hematological disease, chemotherapy administration within 6 months prior to ICU admission, human immunodeficiency virus (HIV) or acquired immunodeficiency syndrome (AIDS), and long-term immunosuppressive treatment (including steroids). Severity was assessed using the Sepsis-related Organ Failure Assessment (SOFA) score, ranging from 0 to 24, with higher scores indicating a higher severity of organ failure (24), at ICU admission and again at HLH diagnosis (defined as the day of bone-marrow aspiration or ferritin measurement). The Delta SOFA score was obtained by subtracting the SOFA score at HLH diagnosis from that at ICU admission.

Patients were classified into four main groups based on HLH etiology: sepsis-related HLH (bacterial, extracellular bacteria, or fungal infection), intracellular infection-related HLH (*Herpesviridae*, COVID-19, influenza, mycobacteria, or pneumocytosis), malignancy-related HLH (active solid cancer or ongoing malignant hemopathy), or idiopathic HLH (patients with acquired and etiologically unexplained HLH). Sepsis-related HLH (extracellular bacteria and fungi) was distinguished from intracellular bacterial infection-related HLH, as the latter leads to different immunological and pathophysiological patterns (25). In addition, intracellular infections are rarely associated with sepsis and are believed to lead to a more specific prognosis. Epstein-Barr Virus (EBV)-associated HLH was retained for patients with a concomitant positive serum EBV polymerase chain reaction (PCR) and HLH diagnosis. Malignancy-related HLH concerned patients who were newly diagnosed with cancer or who relapsed at HLH diagnosis, without concomitant infection. None of the reported cases of HLH were attributed to systemic or auto-immune disease.

After describing the overall population, we compared populations according to their main etiology using the Chi^2^ test (or Fisher exact test) for qualitative variables and analysis of variance (ANOVA–corrected using the Welch method if the variances were in equal between groups) for quantitative variables, followed by a Bonferoni *post-hoc* test.

We analyzed ICU mortality risk factors using univariate and multivariate logistic regression analyses. A 0.20 alpha threshold of significance in univariate analysis was set. Backward variable elimination was then performed to determine factors significantly associated with ICU mortality in multivariate analysis, using a 0.05 threshold for the *p*-value. The main etiology was forced into the model. The Hosmer-Lemeshow test was then used to evaluate the goodness of fit of the model. Quantitative variables are described using medians, with the 25 and 75% interquartile ranges (IQR 25–75%), and qualitative variables using frequencies and percentages. All statistical analyses were performed using SAS 9.4 software. A *p* < 0.05 was considered significant.

## Results

**Figure 1:**
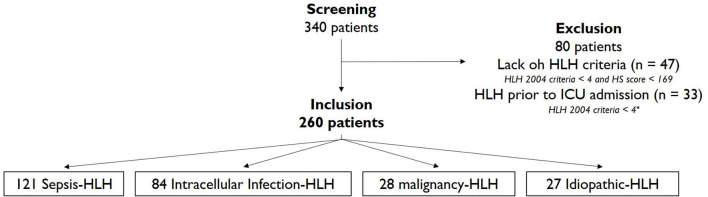
Flow Chart. HLH-2004 criteria < 4 = lack of at least 3 criteria among ferritin, triglycerides, fibrinogen, hemophagocytosis or hepato/splenomegaly.

In total, 260 patients were included from 340 screened files over a 10-year inclusion period (Figure 1). Patient characteristics are presented in Table 1. Excluded patients are described in Supplementary File 1. The median age was 60 years (50–69), with a male predominance (sex ratio 2.25/1). Most of the patients were immunosuppressed (*n* = 165, 64%). The median SOFA score at ICU admission was 9 (7–11). The main HLH trigger was infections: 47% sepsis related-HLH (*n* = 121: 102 extracellular bacteria and 19 fungi—details in Supplementary File 2) and 32% intracellular infection-related HLH (*n* = 84: 59 Herpesviridae, nine influenzae virus, eight COVID-19, four pneumocystis, and four mycobacteria). Malignancy-related HLH accounted for 11% of the cases [*n* = 28: 14 non-Hodgkin lymphoma (NHL), six solid-organ cancers, five acute myeloid leukemia (AML), two EBV-associated NHL, and one multiple myeloma (MM)] and idiopathic HLH 10% (*n* = 27). Among the sepsis-related HLH, 53% enhanced positive viral samples (significant or insignificant viremia, positive buccal or bronchial swabs for Cytomegalovirus (CMV), Herpes Simplex Virus (HSV) and EBV), but only 4% of them had a significant viremia requiring targeted treatment. For these patients, the bacterial trigger was patent and viral replication was secondary. The median HScore was 200 (176–230), corresponding to an 88% (65–98) probability of HLH. A median of five HLH-2004 criteria (4–5) were fulfilled although levels of sIL-2r were available for only 10 patients with a mean of 7362.5 U/mL (825–41177) as shown in Supplementary File 3. Sixteen percentage of the cohort fulfilled the criteria of only one score (HLH-2004 8%, HScore 8%). Bone marrow hemophagocytosis was reported for 91% of patients (*n* = 237). Steroids were used to treat 54% of patients (*n* = 141), whereas other HLH-related treatments (etoposide, cyclosporin A) were exceptionally used. ICU mortality was 57% (*n* = 147), with a median length of stay of 23 days [IQR 13–41]. Six-month survival was 28% (*n* = 72).

**Table 1:** Characteristics of the 260 HLH patients in ICU.

**Patients**	**Number ***n*** = 260**	**Percentage**
Age (years)	60 (50–69)	-
Gender (M/F)	2.25/1	-
**Prior immunosuppression**	165	64
**Severity assessment**		
SOFA at ICU admission	9 (7–11)	-
Delta SOFA	3 (1–5)	-
Invasive MV	139	53
Vasoactive drugs	167	64
RRT	37	14
**HLH etiology**		
Sepsis related-HLH	121	47
Intracellular infections related-HLH	84	32
Malignancy related-HLH	28	11
Idiopathic HLH	27	10
**HLH biology**	-	-
Hemoglobin, g/dL	8.3 (7.6–9)	-
Platelets, G/L	34 (15–58)	-
Neutrophils, G/L	5 (1.7–11.7)	-
Fibrinogen, g/L	4.1 (2.4–6)	-
Ferritin, μg/L	2,677 (1,325–5,191)	-
Triglycerides, g/L	2.6 (1.8–4)	-
LDH, UI/L	493 (311–985)	-
**Bone marrow aspiration**		-
Positive (HmP)	237	91
Negative (no HmP)	13	5
Unavailable	10	4
**HLH diagnostic criteria**		
HScore	200 (176–230)	-
HLH probability with HScore	-	88 (65–98)
HLH-2004 criteria	5 (4–5)	-
**HLH treatment**		
Steroids	141	54
Ciclosporin A	15	6
Intravenous immunoglobulin	17	6
Etoposide	21	8
**Outcomes**		
ICU length of stay, days	23 (13–41)	-
ICU mortality	147	57
6-month survival	72	28

*MV, mechanical ventilation; RRT, renal replacement therapy; SOFA, Sepsis-related Organ Failure Assessment; HLH, hemophagocytic lymphohistiocytosis; Delta SOFA, SOFA at HLH diagnosis – SOFA at admission; LDH, lactate dehydrogenase; HmP, hemophagocytosis; ICU, intensive care unit. Variables are presented in medians with interquartile range of 25% and 75%*.

The clinical and biological criteria according to the main HLH etiology are presented in Table 2. Immunosuppression was more frequent for malignancy-related HLH patients (*n* = 27/28, 96%, *p* < 0.001). Analysis of the ICU severity score showed a significantly higher SOFA score [9 (7–12), *p* = 0.04] for sepsis-related HLH patients. The Delta SOFA did not significantly differ between the main groups. Cytopenia was significantly more pronounced in malignancy-related HLH, with more profound anemia [hemoglobin = 7.9 g/dL (7.5–8.7), *p* = 0.49], neutropenia [neutrophils = 3 G/L (1.3–6.4), *p* = 0.39], and thrombopenia [platelets = 32 G/L (11–49), *p* = 0.03], except when compared to the sepsis related-HLH group. Malignancy-related HLH patients showed markedly higher HLH biomarker levels, with lower fibrinogen [2.4 g/L (1.8–5.2), *p* = 0.001] and higher ferritin levels (5,128 g/L (3029–6842), *p* = 0.14], and subsequently higher HScores [239 (213–270)] and a higher probability of HLH [99% (94–99), *p* = 0.051]. Only four patients lacked the ferritin measurement at HLH diagnosis, but had an HScore > 169 [185 (74.65% probability), 184 (73.5% probability), 182 (70.9% probability), and 223 (96.88% probability)]. When treated, HLH-specific therapy was introduced within a 2-day (1–8) period, the timeline being longer for idiopathic HLH than for the other etiologies [12 (8–16), *p* < 0.001]. Sepsis-related HLH patients received steroids less often than the others [n = 51 (42%), *p* = 0.002] and less anti-proliferative therapy[for etoposide, n = 6 (5%)] than patients with malignancy-related HLH [n = 5 (18%), *p* = 0.13]. Although ICU mortality was similar in patients with malignancy-related HLH [n = 17/28 (61%), *p* = 0.59] and intracellular infection-related HLH [*n* = 51/84 (61%)], the first group showed the highest 6-month mortality rate (82%). Patients with sepsis-related HLH enhanced the lowest mortality rates in both ICU and at 6-month follow-up with respectively 53 and 70% fatality rates.

**Table 2:** Patient characteristics according HLH etiological clusters.

**Patients, ***n***(%)**	**Sepsis-hlh** ***n*** **= 121(47)**	**Intracellular** **Infection-hlh** ***n*** **= 84 (32)**	**Malignancy-hlh** ***n*** **= 28 (11)**	**Idiopathic hlh** ***n*** **= 27 (10)**	***P***
**Immunosuppression**, ***n***(%)	81 (67)	42 (50)	27 (97)	15 (56)	<0.001
None, n	40	42	1	11	-
SOT or HSCT, n	14	10	4	3	-
Ongoing solid cancer, n	29	12	6	5	-
Malignant hemopathy, n	33	12	22	6	-
Recent chemotherapy, n	40	14	8	6	-
HIV or AIDS, n	1	3	1	0	-
Other IS therapies, n	15	8	1	5	-
**Severity assessment**					
SOFA at ICU admission	9 (7–12)	8 (6–11)	8 (6–10)	9 (6–12)	0.04
Delta SOFA	3 (1–4)	4 (1–6)	3 (0–5)	3 (0–5)	0.15
**HLH biology**	-	-	-	-	-
Hemoglobin, g/dL	8.2 (7.6–9)	8.5 (7.7–9)	7.9 (7.5–8.7)	8.4 (7.6–8.8)	0.49
Leukocytes, G/L	6.4 (2.9–14.2)	7.3 (3–18)	4.5 (2.2–7.8)	5.4 (3–11.8)	0.34
Neutrophils, G/L	5.2 (1.6–11.7)	6.5 (2.3–14.6)	3 (1.3–6.4)	5 (2–9.9)	0.39
Lymphocytes, G/L	0.4 (0.1–0.7)	0.5 (0.2–0.8)	0.4 (0.2–0.8)	0.5 (0.1–0.8)	0.87
Platelets, G/L	26 (13–52)	42 (18–68)	32 (11–49)	40 (18–56)	0.03
Fibrinogen, g/L	4.4 (3.3–6.4)	4.1 (2.3–6.1)	2.4 (1.8–5.2)	3.1 (2.1–4.6)	0.001
Ferritin, μg/L	2,518 (1,269–4,271)	2,837 (1,360–5,676)	5,127 (3,029–6,841)	2,325 (997–3,859)	0.14
ASAT, UI/L	62 (36–127)	85 (38–139)	74 (45–210)	51 (38–175)	0.37
ALAT, UI/L	59 (30–111)	51 (34–122)	47 (26–72)	5 (24–221)	0.56
Triglycerides, g/L	2.5 (1.7–3.6)	3 (1.9–4.1)	3 (2–4.3)	2.5 (1.1–4.3)	0.54
LDH, UI/L	461 (289–803)	491 (335–1012)	704 (304-1,136)	843 (346–1,661)	0.37
**HLH diagnostic criteria**					
Bone marrow HmP, *n* (%)	114 (94)	77 (92)	23 (82)	23 (85)	0.34
HScore	199 (175–219)	196 (177–228)	239 (213–270)	190 (171–221)	0.051
HLH probability with HScore, %	88 (64–96)	85 (67–98)	99 (94–100)	80 (55–97)	0.051
HLH-2004 criteria	4 (4–5)	5 (4–5)	5 (4–5)	4 (4–5)	0.79
**HLH treatment**	-	-	-	-	-
Steroids, *n* (%)	51 (42)	51 (61)	19 (68)	20 (74)	0.002
Ciclosporin A, *n* (%)	8 (7)	3 (4)	0 (0)	4 (15)	0.07
IV immunoglobulin, *n* (%)	4 (3)	8 (10)	3 (11)	2 (7)	0.23
Etoposide, *n* (%)	6 (5)	8 (10)	5 (18)	2 (7)	0.13
**Outcomes**					
ICU length of stay, days	23 (14–42)	28 (17–50)	14 (8–28)	24 (10–40)	0,08
ICU mortality, n (%)	64 (53)	51 (62)	17 (61)	15 (56)	0.59
6-month survival, n (%)	36 (30)	24 (29)	5 (18)	7 (26)	0.53

*SOT, solid organ transplant; HSCT, hematopoietic stem cell transplant; HIV, human immunodeficiency virus; AIDS, acquired immunodeficiency syndrome; IS, immunosuppressive; SOFA, Sepsis-related Organ Failure assessment; ICU, Intensive Care Unit; Delta SOFA, SOFA at HLH diagnosis – SOFA at admission; HLH, hemophagocytic lymphohistiocytosis; ASAT, aspartate aminotransferase; ALAT, alanine aminotransferase; LDH, lactate dehydrogenase; HmP, hemophagocytosis. Variables are shown as medians with interquartile range of 25% and 75%*.

The clinical and biological differences between patients according to their binary ICU outcome (alive or deceased) are highlighted in Table 3. Deceased patients were older [65 years (55–71) vs. 55 years (44–64), *p* < 0.001]. The mortality rate for patients receiving renal replacement therapy also tended to be higher (18 vs. 10%, p = 0.07). Death correlated with a higher SOFA score at ICU admission [9 (7–12) vs. 8 (7–11), *p* < 0.001] and a higher delta SOFA [4 (2–6) vs. 2 (0–4), *p* < 0.001]. Laboratory abnormalities associated with ICU mortality included lower hemoglobin values [8.2 g/dL (7.6–8.8) vs. 8.4 g/dL (7.7–9.5), *p* = 0.01], higher LDH levels [545 UI/L (336–1012) vs. 471 UI/L (294–917), *p* = 0.03] and more pronounced hepatitis [ASAT = 76 UI/L (37–214) vs. 57 (36–132), *p* = 0.03; bilirubinemia = 46 μmol/L (21–118) vs. 34 (16–66), *p* = 0.002]. No significant differences were noted for platelet or leukocyte counts or fibrinogen, triglyceride, or ferritin levels between these two groups. A higher number of deceased patients received steroids [*n* = 88/147 (60%) vs. 52/111 (47%), *p* = 0.04], whereas etoposide was equally administered between the groups. Multivariate analysis (Table 4) identified six mortality risk factors in ICU-HLH: older age [OR (5 years) = 1.31 (1.16- 1.48), *p* < 0.0001], higher SOFA score at ICU admission [OR = 1.37 (1.21–1.56), *p* < 0.0001], SOFA score aggravation between ICU arrival and HLH diagnosis (delta SOFA) [OR = 1.47 (1.28–1.70), *p* < 0.0001], presence of bone-marrow hemophagocytosis [OR = 5.27 (1.11–24.97), *p* = 0.04], and more severe anemia [OR = 1.44 (1.09–1.91), *p* = 0.01] and hypofibrinogenemia [OR = 1.21 (1.04–1.41), p = 0.02]. HLH etiology was not an independent risk factor of ICU mortality.

**Table 3:** Differences between HLH patients according ICU mortality- univariate analysis.

**Patients, ***n*** (%)**	**Alive** ***n*** **= 111 (43)**	**Deceased** ***n*** **= 147 (57)**	***P***
Age (years)	55 [44-64]	65 [55-71]	< 0.001
Gender (M/F)	1.64/1	2.87/1	0.04
**Prior immunosuppression**, ***n*** (%)			
**Severity assessment**			
Invasive MV, *n* (%)	54 (49)	84(57)	0.18
RRT, *n* (%)	11 (10)	26(18)	0.08
SOFA at ICU admission	8 (7–11)	9 (7–12)	<0.001
Delta SOFA	2 (0–4)	4 (2–6)	<0.001
**HLH biology**			
Hemoglobin, g/dL	8.4 (7.7–9.5)	8.2 (7.6–8.8)	0.01
Leukocytes, G/L	5 (1.9–13)	7.2 [3.1-14.6]	0.46
Platelets, G/L	35 (15–62)	33 [14-52]	0.62
Fibrinogen, g/L	4.4 (2.5–6.4)	3.9 [2.3-5.6]	0.051
Bilirubin, μmol/L	34 (16–66)	46 (21–118)	0.002
ASAT, UI/L	57 (36–132)	76 (37–214)	0.03
ALAT, UI/L	49 (28–104)	57 (32–135)	0.34
LDH, UI/L	471 (294–917)	545 (336–1,012)	0.03
Ferritin, μg/L	2,704 (1,254–5,191)	2,681 (1,325–5,201)	0.20
Triglycerides, g/L	3 (1.9–4.2)	2.5 (1.7–3.8)	0.09
**HLH criteria**			
Bone marrow HmP, *n* (%)	95 (91)	140 (98)	0.01
HScore	200 (88)	195 (85)	0.867
HLH-2004 criteria	4 (4–5)	5 (4–5)	0.344
**HLH therapies**			
Steroids, *n* (%)	52 (47)	88 (60)	0.04
Etoposide, *n* (%)	6 (5)	15 (10)	0.16

*MV, mechanical ventilation; RRT, renal replacement therapy; SOFA, sequential organ failure assessment; Delta SOFA, SOFA at HLH diagnosis – SOFA at admission; HLH, hemophagocytic lymphohistiocytosis; ASAT, aspartate aminotransferase; ALAT, alanine aminotransferase; LDH, lactate dehydrogenase; HmP, hemophagocytosis. Results are presented in medians, 25% and 75% interquartile ranges*.

**Table 4:** Multivariable analysis: mortality risk factors in ICU acquired adult HLH-logistic regression.

**Risk factors**	**Unit**	**OR [IC95%]**	**p**
HLH etiology (ref=sepsis)			0,54
Others vs sepsis		1.72 [0.82 – 3.57]	0.1499
Cancer vs sepsis		1.39 [0.46 – 4.17]	0.5569
Idiopathic vs sepsis		1.43 [0.46 – 4.39]	0.537
Age	+ 5 years	1.31 [1.16- 1.48]	< 0,0001
SOFA at ICU admission	+1 point	1.37 [1.21-1.56]	< 0,0001
Delta SOFA	+ 1 point	1.47 [1.28-1.70]	< 0,0001
Bone marrow hemophagocytosis	Positive Vs Negative	5.27 [1.11-24.97]	0.04
Hemoglobin	−1 point (g/dL)	1.44 [1.09-1.91]	0.01
Fibrinogen	−1 point (g/L)	1.21 [1.04-1.41]	0.02

*HLH, hemophagocytic lymphohistiocytosis; SOFA, sequential organ failure assessment; ICU, Intensive Care Unit; Delta SOFA = SOFA at HLH diagnosis – SOFA at ICU admission Goodness of fit, adequate (Hosmer and Lemeshow test: p = 0,15)*.

## Discussion

We report one of the largest cohorts of ICU-HLH in adults (26). Our retrospective findings need to be confirmed by further studies, as the number of HLH diagnoses could have been underestimated (potentially incomplete medical coding) or overestimated (low specificity of HLH-2004 and HScore criteria). Besides, the advent of the cytokine era supporting HLH diagnosis (27–29) was not available in most patients of our cohort due to both retrospective design, time of inclusion and parameters unscreened routinely. In addition, retrospective etiological diagnosis can be difficult, as various concomitant etiologies can lead to HLH. Our reported cases are representative of the ICU population and are in accordance with the literature. We confirmed the association between acquired HLH and a poor prognosis, frequent multiorgan failure, and a propensity for a background of immunosuppression. The high ICU mortality rate varied from 50 to 60%, in accordance with both the adult and pediatric literature (26, 30). Mortality risk factors included age and the severity of organ failure assessed by the SOFA score (31–33). The worsening of organ failure prior to the HLH diagnosis led to a worse prognosis (Delta SOFA). The Delta SOFA slightly correlated with a longer time interval between ICU admission and HLH diagnosis (Rho = 0.19, *p* = 0.002). This finding underscores the necessity of a prompt diagnosis and immediate treatment, although ICU-HLH is still widely underdiagnosed according to the literature (34). Persistent fever that is refractory to antibiotics, pancytopenia, major hyperferritinemia, or unexplained liver enzyme elevation should lead practitioners to screen patients for HLH (35–37). Although variable and non-specific, bone-marrow hemophagocytosis is still a hallmark HLH criterion (38–40) and was associated with a higher ICU mortality rate in our study. Among biological factors, non-regenerative profound anemia and hypofibrinogenemia have been reported to be independent predictors of a poor outcome (31–33, 41). The severity of cytopenia and coagulopathy have been shown to correlate with both the prognosis and TNF and IFN-γ-mediated cytokine storm flares (2, 3). High ferritin levels were not significantly associated with ICU mortality, contrary to most of the literature (31, 37) although one article described intensive care requirement in pediatric HLH as a better prognostic factor than ferritinemia (42). Ferritinemia reflects macrophage activation and the IL-1β/IL-18 signaling pathway and is a reliable HLH biomarker for disease follow-up and monitoring, although the 500 μg/L threshold currently used has been shown to have poor sensitivity and specificity in the ICU (36, 43–46).

In our study, mortality was not significantly associated with HLH etiology contrarily to previous reports (10, 11, 30). However, reported mortality was higher for patients with underlying lymphoma or intracellular infection (60%) than for those idiopathic or sepsis-related HLH (50–55%). None of our patients presented with iatrogenic HLH following the administration of CAR T-cells or other immunotherapy (47). Aggressive lymphoma further worsened the poor outcome due to the exacerbation of organ failure (48, 49). Patients admitted to the ICU with intracellular infections (mycobacteria, pneumocystosis, *Herpesviridae*) tended to have a greater mortality rate due to underlying severe comorbidities and/or immunosuppression.

We expected the prognosis to differ according to HLH physiopathology. Lymphocytes can initiate HLH and cytokine storms (lymphoid HLH). The pathophysiology of EBV-associated HLH implies an immune defect that leads to lymphoproliferation (50, 51). The involvement and proliferation of a tumor clone was evident in the pathogenesis of lymphoma-associated HLH. However, lymphoproliferation was not encountered in sepsis-associated HLH. Such patients show enhanced lymphopenia and tissue lymphocyte apoptosis (52, 53). Hence, sepsis-associated HLH would imply a myeloid-induced cytokine storm (myeloid cell-associated HLH). *Toll Like Receptors* have been shown to sustain stimulation in murine models and human inflammasome gain-of-function genetic mutations have been associated with HLH (54–59). Myeloid vs. lymphoid onset thus illustrates two separate pathophysiological mechanisms, highlighting the necessity of better-targeted therapies. Anti-lymphoproliferative treatments, such as etoposide and cyclosporin A, would solely be of interest in lymphoma or EBV-associated HLH, alone or associated with an etiological treatment (rituximab for EBV, specific chemotherapy for lymphoma), although results were not significant in ICU patients (33). These therapies should not be used in sepsis-related and infection-related HLH as they are associated with greater death (60, 61). The most severe and hyperinflammatory cases of sepsis-associated HLH could benefit from therapies that target cytokine storms, such as anakinra (anti-IL-1), tocilizumab (anti-IL-6), and/or ruxolitinib (anti-JAK2) (16, 62, 63).

Our study did not reveal any differences in the prognosis according to the specific administered HLH treatment contrarily to previous reports (12, 33). However, the number of treated patients, especially those treated with cyclosporin A, etoposide, and intravenous immunoglobulin (IVIG), was too low to show any statistical pattern. As IVIG administration is safer than HLH immunosuppressive therapies, its use in non-malignancy-related HLH needs to be studied in a prospective-controlled trial (64). Based on the literature, patients with malignancy-associated HLH and EBV-associated HLH should receive early targeted therapy (33, 65). For other HLH etiologies, the use of steroids has been debated but does not appear to provide any benefit (66). The recently approved anti-IFN-gamma monoclonal antibody (emapalumab) is strictly applicable to only primary HLH and cannot be extended to ICU-HLH (67).

Limitations encompass the retrospective design with a risk of underestimation of HLH incidence in ICU due to imperfect medical coding or overestimation due to scores with low specificity, the absence of a control cohort, and the severity of included patients as they were all admitted in critical care contrary to studies on etoposide efficacy (33).

## Conclusion

Still underdiagnosed, hemophagocytic lymphohistiocytosis was associated with a 50 to 60% mortality rate in the ICU, reaching 70% at the 6-month follow-up, regardless the etiology. The prognosis worsens with the severity of organ failure and cytokine storms. An often non-specific clinical and/or biological set of arguments, above all for immunosuppressed patients, should lead practitioners to search for HLH. Thorough and rapid etiological screening is an absolute priority as it leads to rapid selection of appropriate targeted therapy. As there was no significant difference in mortality with the use of etoposide, a better selection of patients is required for targeted therapy. Prospective studies are needed for a greater understanding of HLH pathophysiology in ICU patients. Secondarily, clinical trials would evidence the best therapeutic strategy for ICU-HLH.

## Data Availability Statement

The raw data supporting the conclusions of this article will be made available by the authors, without undue reservation.

## Ethics Statement

The studies involving human participants were reviewed and approved by Assistance-Publique Hôpitaux de Marseille. Written informed consent for participation was not required for this study in accordance with the national legislation and the institutional requirements.

## Author Contributions

JC devised and supervised the study. AB and JC performed research and wrote the manuscript. AB is the guarantor for the content of the manuscript, including the data, and analysis. VP analyzed the data. AB, JB, JA-S, LP, SH, CG, DM, MG, and JC took care of patients. GK provided key expertise. All authors read and approved the final manuscript.

## Conflict of Interest

The authors declare that the research was conducted in the absence of any commercial or financial relationships that could be construed as a potential conflict of interest.

## Publisher's Note

All claims expressed in this article are solely those of the authors and do not necessarily represent those of their affiliated organizations, or those of the publisher, the editors and the reviewers. Any product that may be evaluated in this article, or claim that may be made by its manufacturer, is not guaranteed or endorsed by the publisher.
