# Supplementary material for: High Mortality of HLH in ICU Regardless Etiology or Treatment
Source: Front Med (Lausanne). 2021 Oct 6;8:735796. doi: 10.3389/fmed.2021.735796 (PMC8526960; doi:10.3389/fmed.2021.735796)
Supplement: Supplementary file 1 [file Data_Sheet_1.docx]

**Additional File 1**. sIL-2r data and NK cell functional analysis.

| NK degranulation / perforin assay | sIL-2r (U/mL) | HLH etiology | Background |
| --- | --- | --- | --- |
| Altered degranulation  Normal distribution of CD56^dim^, CD56^bright^ Decreased CD16 and NCR expression Increased NK CD69+ levels  NK activation | 11000 | Sepsis | Stable metastatic prostate cancer |
| - | 3280 | Sepsis | Stable Crohn’s disease treated with anti-TNF |
| - | 1796 | Sepsis | - |
| - | 4784 | Sepsis | - |
| - | 3491 | Sepsis | - |
| - | 5918 | Sepsis | Bipulmonary transplant for idiopathic pulmonary fibrosis |
| - | 4176 | Viral | - |
| - | 41177 | Viral | Still’s disease |
| - | 825 | Idiopathic | Heatstroke |
| - | 8167 | Idiopathic | Remitted HL treated with HSCT |

*TNF: tumor necrosing factor, HL: Hodgkin lymphoma, HSCT: hematopoietic stem cell transplant*

**Additional File 2**. Characteristics of the 80 excluded patients.

| HLH main etiology | Precise etiology | Age | Sex | Reason for exclusion |
| --- | --- | --- | --- | --- |
| Intracellular infection | Histoplasmosis | 41 | M | HLH prior to ICU admission  (N = 47) |
|  | Strongyloidiasis | 23 | F |  |
|  | EBV | 63 | M |  |
|  | COVID-19 | 71 | M |  |
|  | COVID-19 | 48 | M |  |
|  | COVID-19 | 29 | M |  |
|  | COVID-19 | 23 | M |  |
|  | COVID-19 | 46 | M |  |
|  | Influenza A | 44 | M |  |
|  | Influenza A | 73 | F |  |
| Sepsis | *Fusarium* fungemia | 68 | M |  |
|  | Pneumococcal bacteriemia | 71 | F |  |
|  | Meningococcemia & meningococcal meningitis | 25 | M |  |
|  | Meningococcal meningitis | 51 | M |  |
| Idiopathic | Splenectomy for primary myelofibrosis | 64 | F |  |
|  | ARDS on iatrogenic hemoptysis | 48 | M |  |
|  | ARDS after BPT | 44 | M |  |
|  | ARDS of unknown etiology | 30 | F |  |
|  | PGD on BPT | 34 | M |  |
| Malignancy | Breast carcinoma | 59 | F |  |
|  | NHL | 48 | F |  |
|  | DLBCL | 59 | F |  |
|  | DLBCL | 53 | F |  |
|  | AML | 53 | F |  |
|  | ALL B | 34 | M |  |
|  | Pancreas adenocarcinoma | 57 | M |  |
|  | NHL | 45 | M |  |
|  | AML | 63 | F |  |
|  | AML | 52 | F |  |
|  | NHL | 66 | M |  |
|  | AML | 79 | M |  |
|  | AML | 73 | M |  |
|  | AML | 32 | M |  |
|  | AML | 69 | F |  |
|  | CLL | 43 | F |  |
|  | NHL | 49 | M |  |
|  | AML | 46 | M |  |
|  | ALL | 31 | M |  |
|  | MM | 55 | M |  |
|  | DLBCL | 75 | M |  |
|  | Pulmonary adenocarcinoma | 71 | M |  |
|  | Lymphoblastic lymphoma | 50 | M |  |
|  | AML | 74 | M |  |
|  | ALL | 34 | M |  |
|  | AML | 63 | F |  |
|  | NHL | 60 | M |  |
|  | MM | 55 | M |  |
| Intracellular infection | COVID-19 | 49 | F | Lack of HLH criteria  (N = 33) |
|  | COVID-19 | 50 | M |  |
|  | Influenza B | 46 | M |  |
|  | Influenza A | 44 | M |  |
|  | HSV | 63 | M |  |
| Sepsis | *E.faecalis* bacteriemia | 70 | M |  |
|  | *S.lugdunensis* bacteriemia | 48 | F |  |
|  | *C.difficile* colitis | 64 | M |  |
|  | *E.faecium* bacteriemia | 60 | M |  |
|  | *E.faecalis* bacteriemia | 44 | F |  |
|  | *C.freundii* UTI | 54 | F |  |
|  | *E.coli* bacteriemia | 56 | M |  |
|  | *P.aeruginosa & K.pneumoniae* VAP | 34 | M |  |
|  | *C.albicans* fungemia | 57 | M |  |
|  | *C.albicans* fungemia | 45 | M |  |
| Malignancy | Breast carcinoma | 59 | F |  |
|  | NHL | 49 | F |  |
|  | DLBCL | 70 | M |  |
|  | AML | 54 | F |  |
|  | Sacrum sarcoma | 65 | M |  |
|  | AML | 52 | F |  |
|  | NHL | 52 | F |  |
|  | MM | 51 | M |  |
|  | NHL | 72 | M |  |
|  | DLBCL | 31 | F |  |
|  | AML | 71 | F |  |
|  | AML | 50 | M |  |
|  | MM | 69 | M |  |
|  | CLL | 29 | M |  |
|  | Hodgkin lymphoma | 45 | F |  |
|  | Colon carcinoma | 71 | M |  |
|  | Breast carcinoma | 47 | F |  |
|  | Ovarian cancer | 72 | F |  |

*EBV: Epstein Barr virus, ARDS: acute respiratory distress syndrome, ICU: intensive care unit, BPT: bi-pulmonary transplant, PGD: primary graft dysfunction, NHL: non-Hodgkin lymphoma, DLBCL: diffuse large B cell lymphoma, AML: acute myeloid leukamia, ALL: acute lymphoid leukemia, CLL: chronic lymphoid leukemia, MM: multiple myeloma, HSV: Herpesssimplex virus, UTI: urinary tract infection, VAP: ventilator-associated pneumonia*

**Additional File 3.** Bacteria, mycobacteria and viruses in infection-associated HLH groups.

| Gender | Species | n  N= 102 | % |
| --- | --- | --- | --- |
| **Streptococcus** | *-* | 11 | 11 |
|  | *pneumoniae* | 4 | - |
|  | *Intermedius* | 1 | - |
|  | *Constellatus* | 1 | - |
|  | *Sanguinis* | 1 | - |
|  | *Mitis* | 1 | - |
|  | *Anginosus* | 2 | - |
|  | *Dysgalactiae* | 1 | - |
| **Enterococcus** | *-* | 36 | 35 |
|  | *Faecium* | 15 | - |
|  | *Faecalis* | 16 | - |
|  | *Hirae* | 1 | - |
|  | *Gallinarum* | 1 | - |
|  | *Casseliflavus* | 1 | - |
|  | *Avium* | 2 | - |
| **Staphylococcus** | *-* | 28 | 27 |
|  | *Aureus* | 11 | - |
|  | *Epidermidis* | 9 | - |
|  | *Haemolyticus* | 5 | - |
|  | *Capitis* | 2 | - |
|  | *Lugdunensis* | 1 | - |
| **Other GPB** | *-* | 4 | 4 |
|  | *Bacillus cereus* | 1 | - |
|  | *Corynebacterium striatum* | 2 | - |
|  | *Lactobacillus* | 1 | - |
| **Enterobacteriaceae** | *-* | 65 | 64 |
|  | *Escherichia coli* | 25 | - |
|  | *Klebsiella pneumonia* | 12 | - |
|  | *Enterobacter cloacae* | 9 | - |
|  | *Serratia marcescens* | 4 | - |
|  | *Klebsiella oxytoca* | 3 | - |
|  | *Proteus mirabillis* | 2 | - |
|  | *Morganella morganii* | 2 | - |
|  | *Salmonella spp* | 1 | - |
|  | *Citrobacter koseri* | 1 | - |
|  | *Citrobacter spp* | 1 | - |
|  | *Hafnia halvei* | 1 | - |
|  | *Citrobacter brakii* | 1 | - |
|  | *Proteus vulgaris* | 1 | - |
| **NF GNB** | *-* | 37 | 36 |
|  | *Pseudomonas aeruginosa* | 26 | - |
|  | *Stenotrophomonas maltophila* | 5 | - |
|  | *Pseudomonas putida* | 2 | - |
|  | *Acinetobacter pitii* | 2 | - |
|  | *Pseudomonas mosselii* | 1 | - |
|  | *Achromobacter spp* | 1 | - |
| **Other GNB** | *-* | 13 | 13 |
|  | *Legionella* | 2 | - |
|  | *Chryseobacterium* | 1 |  |
|  | *Capnocytophaga* | 1 | - |
|  | *Haemophilus influenza* | 1 | - |
|  | *Branhamella catarrhalis* | 1 | - |
| **Anaerobic bacterio** | *-* | 13 | 13 |
|  | *Clostridium difficile* | 4 | - |
|  | *Fusobacterium necrophorum* | 2 | - |
|  | *Egghertella lenta* | 2 | - |
|  | *Bacteroides vulgatus* | 1 | - |
|  | *Bacteroides spp* | 1 | - |
|  | *Bacteroides ovatus* | 1 | - |
|  | *Clostridium inoculum* | 1 | - |
|  | *Fusobacterium multiforum* | 1 | - |
| **Viruses** | *Cytomegalovirus* | 50 | - |
|  | *Epstein Barr virus* | 36 | - |
|  | *Herpes simplex virus* | 30 | - |
|  | *Human Herpes virus 6* | 13 | - |
|  | *Varicella zoster virus* | 1 | - |
|  | *Rhinovirus* | 4 | - |
|  | *COVID-19* | 8 | - |
|  | *Influenza* | 9 | - |
|  | *BK virus* | 1 | - |
| **Mycobacteria** | *Mycobacterium tuberculosis* | 3 | - |
|  | *Mycobacterium lentiflavum* | 1 | - |

*GPB: Gram-positive bacteria; GNB : Gram-negative bacteria; NF GNB: non-fermenting Gram-negative bacteria*

HLH specific etiology in sepsis group showed an 84.3% bacterial HLH cause, mainly represented by infectious pneumonia.

Bacterial findings enhanced a majority of Enterobacteriaceae 64% infections led by *Escherichia coli* and *Klebsiella pneumoniae*.

Non-fermenting gram negative bacteria 36% (mainly *Pseudomonas aeruginosa,* and scarce *Stenotrophomonas* maltophila) followed in term of frequency, along with enterococcus species 35% (*Enteroccus faecalis & faecium* being equally represented).

*Staphyloccus* infections 27% mainly reported *Staphylococcus aureus* then *Staphylococcus epidermidis*. Streptococci 11% and anaerobic 13% infections were also documented. 61% of bacteria-associated HLH enhanced a polymicrobial documentation.

Viral findings showed 69.5% CMV infection, 45.7% EBV infection, 35.6% HSV infection and 22% HHV6 infection. 61% of patients had at least two concomitant documented viral infections.
